# How culture shapes choices related to fertility and mortality: Causal evidence at the Swiss language border

**DOI:** 10.1017/ehs.2024.19

**Published:** 2024-04-12

**Authors:** Lisa Faessler, Rafael Lalive, Charles Efferson

**Affiliations:** Faculty of Business and Economics, University of Lausanne, Lausanne, Switzerland

**Keywords:** gene–culture coevolution, social learning, cultural variation, cultural border, regression discontinuity design

## Abstract

Results from cultural evolutionary theory often suggest that social learning can lead cultural groups to differ markedly in the same environment. Put differently, cultural evolutionary processes can in principle stabilise behavioural differences between groups, which in turn could lead selection pressures to vary across cultural groups. Separating the effects of culture from other confounds, however, is often a daunting and sometimes intractable challenge for the working empiricist. To meet this challenge, we exploit a cultural border dividing Switzerland in ways that are independent of institutional, environmental and genetic variation. Using a regression discontinuity design, we estimate discontinuities at the border in terms of preferences related to fertility and mortality, the two basic components of genetic fitness. We specifically select six referenda related to health and fertility and analyse differences in the proportion of yes votes across municipalities on the two sides of the border. Our results show multiple discontinuities and thus indicate a potential role of culture in shaping stable differences between groups in preferences and choices related to individual health and fertility. These findings further suggest that at least one of the two groups, in order to uphold its cultural values, has supported policies that could impose fitness costs on individuals relative to the alternative policy under consideration.

## Graphical abstract

**Figure:**
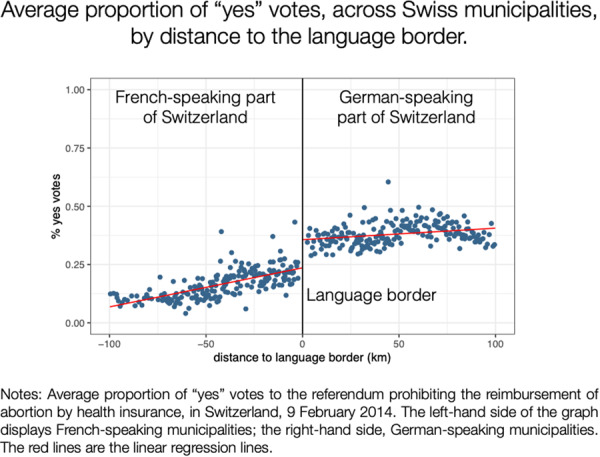

**Social media summary:** Discontinuities at a language border in Switzerland show that culture can shape choices related to health and fertility.

## 1. Introduction

Gene–culture coevolutionary theory argues that human populations are subject to two evolutionary processes, genetic and cultural (Laland, 2008). Genetic variants influence the development and spread of cultural traits, while cultural practices affect selection on genes. As a result, genes and culture coevolve as linked dynamic processes. As a kind of corollary hypothesis, an especially controversial claim is that social learning stabilises cultural differences at the group level, which in turn is a necessary but not sufficient condition for any kind of selection at the level of the cultural group (Henrich, 2004; Richerson et al., 2016).

We examine a kind of proof of concept for these ideas. Specifically, we do not directly consider culture's influence on genetic fitness, but we do insist on an attempt to identify cleanly the causal influence of culture on decisions affecting health and fertility. Identifying cultural variation as a group-level phenomenon is often a difficult empirical challenge because culture typically covaries with many other variables related to institutions, the environment and possibly even genes. To meet this challenge, we exploit a distinctive feature of Switzerland's geography, a linguistic and cultural border that separates the German-speaking part of the country from the French-speaking part. Right at the border, the environments for French speakers and German speakers are necessarily identical. Moreover, the French- and German-speaking parts of the country are genetically similar in general (Buhler et al., 2012). Finally, in some regions, the border does not match any institutional boundary. Thus, right at the border, we have the possibility of observing variation in preferences and norms that we can say is cultural in the precise sense that it cannot be institutional, environmental or genetic. This situation represents an unusual opportunity because cultures often covary with one or more of these variables.

Consider two examples that illustrate the challenges of isolating culture in domains that could influence selection on genes. First, lactase persistence is a classic example. In most mammals, including humans, lactase production declines after weaning, but some populations have evolved the ability to produce lactase throughout adulthood, a condition known as lactase persistence. This adaptation is thought to have arisen in response to the cultural practice of dairy farming, which allowed people to consume milk and dairy products as a significant part of their diet. Nonetheless, recent evidence suggests that multiple factors, including different environmental conditions, have contributed to lactase persistence, and that dairying alone is probably insufficient to explain the spread of the trait. In particular, exposure to famine and diseases has played a crucial role in the evolution of lactase persistence (Evershed et al., 2022).

Second, the cultural practice of cooking and its influence on human gut size is another classic example. Cooking allows us to pre-digest our food over the campfire or on the stove, which improves the biological availability of the nutrients in the food. Cooking as a cultural innovation probably allowed our ancestors to evolve smaller guts because they were able to extract more energy from their food for a given metabolic cost (Wrangham & Carmody, 2010). Thus, energetic resources within the body became available for other functions such as brain growth and development. This shift in energy allocation is thought to have played a key role in the evolution of larger brains and shorter digestive tracts in humans compared with our primate relatives (Navarrete et al., 2011). However, cooking is one of the few human cross-cultural universals. There is no such thing as a human group that does not engage in cooking. Therefore, establishing a causal link between the cultural practice of cooking and alterations in the human gut remains impossible, given the absence of a counterfactual. Stories of this sort are interesting and compelling, and they may very well be correct. They are not, however, causal explanations. Valid comparisons that we could rely on to represent the counterfactual state are not available to us and probably never will be.

### 1.1. Identifying culture

Identifying the causal influence of culture on gene selection is a challenge. Comparing the average behaviours of two populations (Bell et al., 2009) often cannot provide evidence for cultural variation. If environmental conditions, institutions and other socioeconomic variables covary with culture, isolating the extent to which group-level variation is specifically cultural can be exceedingly difficult. Lamba and Mace (2011), for example, compared groups within the same culture but living in different locations, and they found substantial variation across the groups. This kind of result suggests that large differences among groups can be environmental just as surely as they can be cultural, and indeed recent evidence suggests that ecology can explain a substantial amount of human population diversity (Wormley et al., 2022).

That said, a number of new tools have been developed to allow the identification of causal effects without randomised experiments, and these tools can potentially help us identify culture. These quasi-experimental methods include the regression discontinuity design. The basic idea of the regression discontinuity design is to compare the outcomes of individuals just above and below some threshold. Intuitively, researchers estimate two regression lines, one on each side of the threshold, and doing so identifies any discontinuities in the response variable that occur right at the threshold (Cattaneo et al., 2019; Lee & Lemieux, 2010). A few studies have used a variant of this method, the spatial regression discontinuity design, to identify cultural discontinuities and the Swiss language border. We adopt the same basic approach here.

These studies are known as ‘Röstigraben studies’, a type of spatial regression discontinuity design that examines cultural differences in behaviour in Switzerland. The term ‘Röstigraben’ – German for ‘hash brown trench’ – refers to a linguistic and cultural border within Switzerland. The border separates the German-speaking part from the French-speaking part of the country, and in some regions it does not match any institutional boundary. With appropriate data, researchers could in principle check for discontinuities in any variable of interest right at the language border, and by doing so the researcher would effectively isolate cultural differences, as a group-level phenomenon, in identical institutional and ecological settings. Using this technique, Eugster et al. (2011) document a persistent difference in the demand for social insurance at the border, and Eugster et al. (2017) also found a significant discontinuity in unemployment duration. Focusing on the bilingual canton of Fribourg, Brown et al. (2018) discovered a systematic difference in the financial literacy of students across the border, and their analyses suggest that the effect is driven by cultural differences rather than unobserved heterogeneity in policies.

### 1.2. Switzerland's linguistic and cultural landscape

Switzerland is a multilingual country with four official languages: German, French, Italian and Romansh. German is the most widely spoken language at home (62%), while French is second (22.8%). Switzerland's linguistic diversity is a unique feature that has played a significant role in shaping its culture and society. Multilingualism is a common characteristic among Swiss people. However, the historical border between the French- and the German-speaking regions has remained clear-cut. A sharp change in the main language spoken at home persists when switching from one side of the border to the other (OFS, 2022a). Because the language border is clear and well-defined in space, we can meaningfully isolate discontinuous differences that occur right at the border.

Beyond language, conventional wisdom posits that this linguistic border also captures differences in values, norms and preferences. Swiss media and citizens often view it as a cultural divide that marks contrasting attitudes. During federal elections, when voting on shared issues, these differences become especially apparent (Etter et al., 2014). Furthermore, the French- and German-speaking regions show distinct patterns of health-related behaviours *on average*. For instance, French speakers typically consume more red meat but less butter, milk and coffee than their German-speaking counterparts (Chatelan et al., 2017; Rochat et al., 2019). These comparisons of group averages do not provide causal evidence, but they do fit with the conventional wisdom within Switzerland. When you cross the Röstigraben, it's not just the language that changes; culture more broadly changes too. That said, we can check to see if this is the case with a spatial regression discontinuity design. The basic idea is to code variables of interest as a function of distance from the language border, and then use the method to estimate any discontinuities in the variables right at the border. Doing so is effectively like comparing what happens 1 metre to the east of the border with what happens 1 metre west of the border.

### 1.3. The cultural components of fitness

Having explained our strategy to isolate culture's causal effect, we now turn to the second consideration, namely, what kinds of available data connect possible cultural differences within Switzerland to fertility and mortality, the two basic components of genetic fitness? In our study, we focus on the tendency of people to vote for or against policies that should impact either the survival or reproduction of individuals. In Switzerland, the leading causes of death are predominantly disease. In 2018, cardiovascular diseases contributed to 31% of the deaths, while cancer accounted for 26%. Dementia is third at 10%. Because the majority of deaths are related to (the absence of) health, we focus on choices related to health to understand how culture could influence survival rate. Specifically, we investigate choices related to the healthcare system and the management of pandemics.

Shifting to fertility and drawing on Hrdy's work on the evolutionary basis of parenthood (1999), we focus on women's freedom of choice regarding investments in offspring. Human infants are highly resource-intensive, and raising a human child requires cooperation among multiple caregivers. Humans are cooperative breeders, and presumably women have long been subject to selection for the ability to assess the social support available for raising a child. If adequate support is lacking, women may choose not to invest in the child and prioritise potential future offspring instead. In terms of genetic fitness, women need the freedom to manage trade-offs between investing in current offspring vs. conserving resources for potential future offspring. In that sense, cultural practices that limit women's autonomy could be viewed as imposing a detrimental effect on the fitness of women who have not completed reproduction and on the inclusive fitness of any genetic relatives. We investigate potential differences in support for three types of policy that should influence women's freedom of choice and degree of social support during and after pregnancy. These three types of policy pertain to abortion access, assisted reproduction and paid parental leave.

### 1.4. Discontinuities in voting behaviours and fitness costs

We would like to explain the generic argument for why discontinuities in these voting behaviours are interesting from a gene–culture coevolutionary perspective. First, we select referenda regarding health and reproduction policies that affect fitness through their implications on mortality and fertility. Some policies might favour more children and other policies fewer children. This would mean, in turn, that policies, if enacted, would vary in terms of how they incentivise individuals to manage the trade-offs between the quantity and quality of their offspring. Analogously, some policies might augment the scope for individuals to rely on social support when raising offspring, while other policies might do the opposite. In this way, if enacted, policies would vary in terms of how they incentivise individuals to manage the trade-offs between current and future offspring. Lastly, policies related to pandemics should affect the risk of infectious disease and by extension the risk of mortality. Policies related to healthcare more broadly should affect the extent to which individuals invest in their health and in turn survival. For example, one of the referenda below concerned how to organise health insurance. Even if we imagine that the alternatives would have no consequences in terms of the quality of healthcare supplied, we can easily imagine that different insurance schemes would affect behaviour on the demand side. Some schemes might incentivise healthy lifestyles and preventative treatments, while other schemes might tip the balance in favour of treating people after they get sick.

Second, we estimate potential differences in voting behaviour at the language border. Right at the border, we assume that, among the policies under consideration, one policy is better than the other on average in terms of expected fitness. By this, we do not mean that one policy is best or optimal in absolute terms. Rather, we mean that, between the policies for which citizens vote, one is better than the other. We call this the ‘better’ policy, and our working assumption is that this better policy is the same on both sides of the border. This is a fundamental assumption for our approach. The assumption might be wrong, of course, but focusing on discontinuities right at the border maximises the chances that it is correct. In any case, our task is to examine both the implications and the limitations of this assumption.

This assumption does not mean that the same policy is better than the other throughout all of French- and German-speaking Switzerland. It simply means that the better policy is the same immediately to the west and immediately to the east of the border. In addition, this assumption does not mean that one policy is better for the expected fitness of every individual. Instead, we assume that one is better than the other on average in terms of the expected fitness of individuals in the group. We do not deny individual heterogeneity. Instead, by using the regression discontinuity design and the linguistic border, we aggregate individual differences and focus on the average outcome at the group level.

We do not know which policy is better, nor does the answer to this question matter for present purposes. We simply assume that at the border one is better than the other in terms of average expected fitness. If this is true, then a discontinuity implies that at least one of the two groups does not favour the best of the two policies for cultural reasons, where cultural reasons, by this account, must be separate from institutions, genes and environment. If enacted, the inferior policy would bring an expected fitness cost, however small, on some individuals of the group relative to the other policy under consideration.

Nonetheless, one can challenge the assumption that right at the border one policy is better than the other in fitness terms. We would like to highlight two possibilities. First, in high-dimensional choice spaces with a complex fitness topography, multiple optima can easily exist. Two groups can thus favour two different policies, both of which are roughly equivalent local optima. The two optima in question may or may not be globally optimal. Regardless, the point is that the two policies differ in the details, but they are extremely similar in terms of ultimate outcomes. Second, by examining discontinuities at the border, our approach controls for institutional, geographic and genetic variation as potential confounds. It does not, however, control for all sources of social variation. People living on the two sides of the border may have different social networks, which could lead the value of a given policy to vary as we move from one side of the border to the other. Such scenarios could challenge our assumption that right at the border, on both sides of the border, one single policy is better than the other in terms of average fitness. We cannot definitively rule out such scenarios, but we should reduce the probability that they play a large role precisely because we limit attention to discontinuities.

In sum, our study aims to investigate the causal influence of culture on health- and fertility-related choices and to discuss how any differences might relate to genetic fitness. To meet this goal, we use a quasi-experimental design based on distance from the Röstigraben, a linguistic and cultural border in Switzerland. We are looking for discontinuities in choices at the border. Any discontinuities at the border would suggest a cleanly identified cultural difference that shapes preferences and behaviour. We will then discuss, somewhat speculatively, how these cultural differences could affect the relative fitness of individuals in the two cultural groups.

## 2. Methods

### 2.1. Referanda data

To explore potential cultural differences in decision-making domains related to fertility and mortality, we use data from referenda in Switzerland. The use of a regression discontinuity design necessitates a substantial amount of geographically precise data, which the referenda data provide. We focus on referenda held at the Swiss level and thus common to all cantons. Importantly, referenda occur multiple times a year and encompass a wide range of topics, including health, the healthcare system and fertility. However, our sample represents only the voting population and excludes non-voters’ opinions on both sides of the border. Nonetheless, the laws are based on the decisions of voters. As such, even though our data are not fully representative of the entire Swiss population, they can help identify cultural differences in the voting population. Further, our data are aggregated at the municipal level, rather than at the individual level, presenting a notable limitation in assessing the impact of cultural differences on individual fitness within the two groups. A more direct assessment would involve individual-level data. However, the requirements of our study for large datasets with precise geographical accuracy, combined with the sensitive nature of voting, health and fertility data, ensure that access to individual-level data is strictly limited. Therefore, we have employed municipal-level data as a feasible and effective solution.

We use the percentage of ‘yes’ votes by municipality in referenda as our response variables, and we estimate discontinuities in referenda results across municipalities on both sides of the border. Our analysis focuses on a preregistered list of referenda related to health or fertility in the past decade (Faessler et al., 2022). The data are provided by the Federal Statistical Office and include referenda results across municipalities, with our unit of analysis being the municipality. We selected municipalities within 100 km of the language border, totalling 1409 municipalities.

### 2.2. Regression discontinuity design

A regression discontinuity design has three essential elements: a threshold, a running variable and a treatment. In our case, the threshold is the cultural border, the continuous variable is the distance from this border and the treatment is the culture. Starting from these elements, we estimate two regression lines on each side of the border to examine whether voting results are discontinuous at the border. As such, we study the effect of moving from one side of the language and cultural boundary to the other on referenda outcomes and the distribution of policy preferences these outcomes represent.

The generic regression model for these regression discontinuity designs can be represented as follows:1
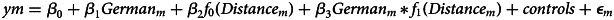


In detail, *y_m_* denotes the outcome of interest for municipality *m*, which is the proportion of ‘yes’ votes for a referendum. *German_m_* is a dummy variable that takes the value of 1 if the municipality is on the German side of the border and 0 otherwise. In that sense, *β*_1_ captures the discontinuity of interest, the cultural discontinuity at the border. A significant *β*_1_ value indicates a causal effect of culture on voting decisions at the border. *Distance_m_* is the running variable that measures the distance from the border. *f*_0_() and *f*_1_() are functions of distance to the border that will be estimated. Both *Distance_m_* and its interaction with *German_m_* take care of controlling for effects that happen away from the border and that could be driven by environmental differences. Throughout the study, we will estimate different versions of this generic regression discontinuity model, each of which will focus on a distinct referendum.

In this analysis, municipality language *German_m_* and distance from the language border *Distance_m_* are our main independent variables. Distance from the border, in particular, plays a crucial role, and we explain in detail how the measure is constructed. First, using the same distance data as Eugster et al. (2011), each municipality is assigned a language according to the language spoken by most of its population. Second, the distance to the language border is calculated by determining the shortest road distance between the focal municipality and the nearest municipality where the other language is spoken. Further, the distance is set as negative for French-speaking municipalities and positive for German-speaking municipalities.

Our statistical model controlled for municipality type because rural and urban areas could exhibit different voting patterns. We control for this possibility by including a dummy for municipality type, i.e. whether the municipality is located in an urban or rural area. We also include canton fixed effects. In Switzerland, a federal system divides power between the state and the cantons. Cantons are administrative subdivisions of the country and have authority over education, healthcare, policing and taxation. In particular, institutions related to health and fertility may vary across cantons. By incorporating a canton fixed effect, we address disparities among cantons and restrict our analysis to variations within each canton. Nevertheless, the language border crosses some cantons and does not correspond to an institutional boundary for this part.

A fundamental assumption of regression discontinuity design is that at the threshold, the treated and control groups differ only by treatment. Because our unit of analysis is the municipality, we necessarily move from one municipality to another at the threshold. However, while municipalities have a certain degree of autonomy, their powers are limited by cantonal and federal laws. Municipalities are mainly responsible for local governance, including waste management, water supply, social welfare and public transport. Thus, even though institutions change from one municipality to another, the institutional changes are limited and not directly related to health and fertility.

Aside from institutions, population characteristics may also vary at the border. Table 1 provide the statistics for a selection of population and municipality variables likely to influence choices related to health and fertility. The variables include population size and characteristics, age structure within the population and a series of wealth indicators. Most of the variables are not perfectly balanced at the border, but regions are more balanced at the border than overall (column ‘Difference’ has larger differences than ‘At the border’). In particular, the age structure and wealth seem to differ on the border's two sides. The municipalities on the German-speaking side count more older individuals and fewer younger individuals while having higher revenues and smaller tax ratios. These differences could suggest that the population on the German-speaking side of the border is more preoccupied with health, but it also benefits from higher revenues to prevent disease or provide medical care.

**Table 1.** Municipalities and population characteristics around the border

	Mean all	French language	German language	Difference	At the border
Population size	3163.38	2609.39	3576.18	966.79	*−*796.118
Population variation (%)	8.83	13.36	5.45	*−*7.91^∗∗∗^	*−*6.784^∗∗∗^
Density	323.73	203.00	413.69	210.69^∗∗∗^	126.498
Immigrants (%)	14.19	16.14	12.74	*−*3.40^∗∗∗^	*−*5.219^∗∗∗^
Average household size	2.31	2.37	2.27	*−*0.10^∗∗∗^	−0.016
0–19 years (%)	20.45	21.97	19.32	*−*2.65^∗∗∗^	*−*2.184^∗∗∗^
20–64 years (%)	59.74	59.75	59.73	*−*0.02	0.303
+65 years (%)	19.81	18.28	20.95	2.67^∗∗∗^	1.882^∗∗∗^
Young dependency ratio	34.52	36.81	32.81	*−*4.00^∗∗∗^	−3.571^∗∗∗^
Mean taxable revenue	69,762	66,617	72,275	5658^∗∗∗^	6079^∗^
Tax rate for families	5.30	4.94	5.57	0.63^∗∗∗^	*−*0.182^∗∗∗^
Tax rate for singles	15.40	15.33	15.45	0.12	*−*0.373^∗∗∗^
Social assistance (%)	2.69	2.80	2.61	*−*0.19	*−*1.087^∗∗∗^

Notes: ‘Mean all’ refers to the mean of municipalities within 50 km of the language border. ‘French language’ includes only the municipalities where most of the population speaks French, within a 50 km range. ‘German language’ refers to the municipalities where most of the population speaks German, within a 50 km range. ‘Difference’ shows the mean difference between French-language municipalities and German-language municipalities. ‘At the border’ shows the difference estimated at the language border using regression discontinuity design and controlling canton, and whether the municipality is urban or rural. † *p* < 0.1; * *p* < 0.05; ** *p* < 0.01; *** *p* < 0.001. Source: Swiss Federal Statistical Office. Distances from search.ch.

To ensure that our results are not influenced by these disparities across French- and German-speaking municipalities, we incorporate additional municipality-level controls in a robustness analysis (presented as model (4) in the results regression tables). Specifically, we account for age structure differences by introducing the following variables: the proportion of individuals below 19 years old, those exceeding 64 years old, the youth dependency ratio, the birth rate and the average household size. Additionally, we address wealth disparities by integrating the average taxable revenue and the tax rates for families and singles. Because we do not know if some of these variables are influenced by culture, we do not treat the analysis that includes these variables as our baseline analysis. Instead, we treat the analysis with these additional variables as a robustness validation, even though some of the variables could be colliders.

Importantly, our setting cannot exclude some individuals deciding to move to the other side of the border. If so, people will self-select their treatments, which will undermine to some extent our identification strategy. While people could decide to live in the region that best matches their values, the language border is sharp. Our data indicate that the mean proportion of French speakers shifts from 74% to 12% within a distance of only 6 km. Similarly, the proportion of German speakers shifts from 24% to 86%. Moving to another linguistic region would require the individual to learn the other language with a fluency level comparable with speaking a language at home, which necessarily constitutes a barrier. Further, the average moving distance for Switzerland is 13 km, and most of the moves (58%) happen within a distance of 5 km (OFS, 2022b). Although we cannot exclude that some individuals self-select in treatments, we suspect that this mechanism has limited effects.

## 3. Results

Our results show multiple discontinuities in voting behaviours at the language border. Of those significant discontinuities, three are related to fertility and one is related to health. These discontinuities suggest the potential for culture to create stable differences between groups in domains related to health and fertility, where cultural explanations are distinct from institutional, genetic and environmental explanations. For each of the referenda, we further speculate how these cultural differences could affect the relative fitness values of individuals in the two cultural groups.

### 3.1. Health-related referanda

#### 28 September 2014, the referendum for a single public health insurance company

First, we analysed the results of the referendum on creating a single public health insurance company, which took place on 28 September 2014. Under the proposed single-payer system, a public insurance company would have replaced the current private insurance companies, and all residents would have been required to enrol in the public plan. Supporters argued that the single-payer system would reduce administrative costs and improve access to healthcare. At the same time, opponents claimed that it would lead to longer waiting times and lower quality of care.

**Figure 1.**
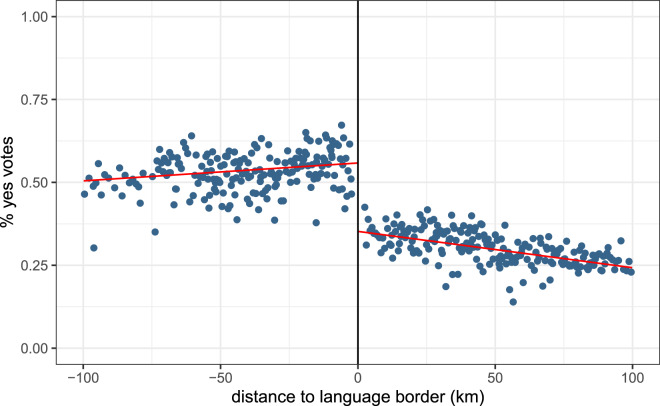
Average proportion of ‘yes’ votes to the referendum for a single public health insurance, by distance to the language border. Notes: The left-hand side of the graph displays French-speaking municipalities; the right-hand side displays German-speaking municipalities. The red lines are the linear regression lines. Source: Federal Statistical Office. Distances from search.ch.

Figure 1 shows a strong discontinuity at the border in the pattern of ‘yes’ vote proportions across municipalities. The left-hand side of the graph displays French-speaking municipalities, while the right-hand side shows German-speaking municipalities. In almost all municipalities on the French-speaking side of the border, the proportion of ‘yes’ votes is higher than in municipalities on the German-speaking side. The red lines represent linear regression lines. Linear regression results in Table 2 confirm the presence of a discontinuity in voting results at the border (estimate = *−*0.221, *p <* 0.001). Further, the German language estimate is not sensitive to controlling for additional municipality-level controls.

**Table 2.** Referenda for a single public health insurance company: regression analysis at the language border

	(1)	(2)	(3) Baseline	(4)
German language	*−*0.199^∗∗∗^	*−*0.219^∗∗∗^	*−*0.221^∗∗∗^	*−*0.209^∗∗∗^
	(0.009)	(0.009)	(0.009)	(0.009)
German × Distance	*−*0.002^∗∗∗^	*−*0.002^∗∗∗^	*−*0.002^∗∗∗^	*−*0.002^∗∗∗^
	(0.0002)	(0.0002)	(0.0002)	(0.0002)
Distance	0.001^∗∗∗^	0.001^∗∗∗^	0.001^∗∗∗^	0.001^∗∗∗^
	(0.0001)	(0.0001)	(0.0002)	(0.0001)
Urban			0.022^∗∗∗^	0.021^∗∗∗^
			(0.004)	(0.004)
Constant	0.558^∗∗∗^	0.574^∗∗∗^	0.554^∗∗∗^	0.766^∗∗∗^
	(0.007)	(0.016)	(0.016)	(0.056)
Cantons FE	No	Yes	Yes	Yes
Municipality controls	No	No	No	Yes
Observations	1,409	1,409	1,353	1,353
Adjusted *R*^2^	0.662	0.804	0.807	0.819

Notes: The regression analysis shows the impact of switching from the French-speaking side of the border to the German-speaking side on voting results, that is, the proportion of ‘yes’ votes in a municipality. ‘German language’ indicates that the primary language of a municipality is German and is our variable of interest. ‘Distance’ is the road distance to the language border. ‘Distance’ and its interaction with ‘German language’ control for effects that happen away from the border and environmental differences. We restrict our analysis to municipalities within 100 km of the language border. Models (2), (3) and (4) include controls for the canton, referred to as Fixed Effects (FE). Models (3) and (4) include a control variable for municipality characteristics, whether the municipality is located in a rural or urban area. Model (4) includes additional controls at the municipality level. Controls include population age structure, average household size, birth rates, average revenue and tax rates. Robust standard errors are in parentheses. † p < 0.1; * p < 0.05; ** p < 0.01; *** p < 0.001. Source: Federal Statistical Office. Distances from search.ch.

The referendum on a single health insurance company highlights an interesting example of the potential influence of culture on fitness. Swiss citizens were asked if they would like a single public health insurance system or multiple private health insurance companies. To illustrate the significance of this choice, imagine two extremes. At one extreme, a single insurance company would pool risk over the entire Swiss population. At the other extreme, each individual would self-insure and be responsible for her own healthcare and associated costs. Whatever the details, the best system in terms of an individual's health, survival and fitness for the people at the border must lie between these two extremes. We observed that the two groups supported different policies at the border. If, however, the better policy on average is the same right at the border, the discontinuity in preferences at the border means that at least one of the two groups supported, for cultural reasons, a worse policy in terms of expected fitness compared with the other policy.

#### 22 September 2013, revision of the law on epidemics and 13 June 2021, Covid law

The second example comes from two referenda related to the management of epidemics. The two referenda are 8 years apart. On 22 September 2013, Switzerland held a first referendum on revising the law on epidemics, and the proposed changes aimed to enhance the country's response to any future pandemics. The revised law would have expanded the government's powers to contain outbreaks, require vaccinations and collect health data for public health reasons. However, groups such as anti-vaxxers and privacy advocates were concerned about the increased surveillance and data collection that could follow. 8 years later, on 13 June 2021, Swiss citizens voted on a related question, namely the Covid law. The proposal was to give the government extraordinary powers to manage the Covid-19 pandemic, such as imposing restrictions on public life and providing financial aid to those affected. However, the law faced opposition from groups who believed that it gave the government too much power and infringed on individual freedoms. A majority vote of around 60% approved both laws.

**Figure 2.**
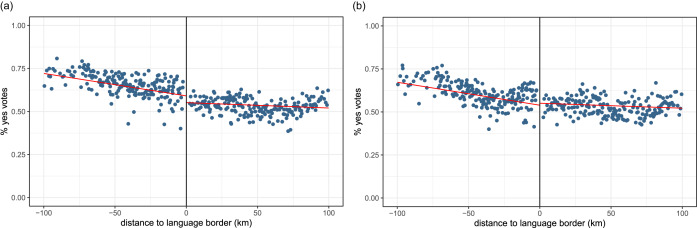
Average proportion of ‘yes’ votes to the two referenda on epidemics management across municipalities, by distance to the language border. (a) The revision of the epidemics law. (b) The Covid law. Notes: The left-hand side of the graph displays French-speaking municipalities; the right-hand side displays German-speaking municipalities. The red lines are the linear regression lines. Source: Federal Statistical Office. Distances from search.ch.

Figure 2 plots the average proportion of ‘yes’ votes for these two referenda across municipalities on the two sides of the language border. The two figures present similar patterns, namely a negative slope on both sides and a steeper slope on the French side. However, these two graphs by themselves do not allow us to confirm or disconfirm the presence of discontinuities at the border.

Tables 3 and 4 present the results of the regression analyses. Both results show quantitatively small estimates whose significance varies across models. In 2013, the municipalities on the German-speaking side of the border were less likely to accept the law, but the difference is not significant in the baseline model (estimate = *−*0.016, *p <* 0.1). In 2021, the effect goes in the opposite direction. Municipalities on the German-speaking side of the border are more likely to vote ‘yes’ (estimate = 0.027, *p <* 0.05). However, the significance disappears in model (4) with the addition of controls at the municipality level. These results should be interpreted with caution, and we treat them as neither significant nor robust. Further analyses are needed to understand whether culture can influence pandemic-related behaviours.

**Table 3.** Revision of the epidemics law: regression analysis at the language border

	(1)	(2)	(3) Baseline	(4)
German language	*−*0.050^∗∗∗^	*−*0.014	*−*0.016^†^	*−*0.026^∗∗^
	(0.009)	(0.010)	(0.010)	(0.009)
German × Distance	0.001^∗∗∗^	*−*0.0003	*−*0.0004^†^	*−*0.0005^∗^
	(0.0002)	(0.0002)	(0.0002)	(0.0002)
Distance	*−*0.001^∗∗∗^	*−*0.001^∗∗∗^	*−*0.001^∗∗∗^	*−*0.0004^∗^
	(0.0001)	(0.0002)	(0.0002)	(0.0002)
Urban			0.062^∗∗∗^	0.048^∗∗∗^
			(0.004)	(0.004)
Constant	0.592^∗∗∗^	0.695^∗∗∗^	0.639^∗∗∗^	0.826^∗∗∗^
	(0.007)	(0.018)	(0.018)	(0.060)
Cantons FE	No	Yes	Yes	Yes
Municipality controls	No	No	No	Yes
Observations	1,409	1,409	1,353	1,353
Adjusted *R*^2^	0.382	0.525	0.594	0.635

Notes: The regression analysis shows the impact of switching from the French-speaking side of the border to the German-speaking side on voting results, that is, the proportion of ‘yes’ votes in a municipality. ‘German language’ indicates that the primary language of a municipality is German and is our variable of interest. ‘Distance’ is the road distance to the language border. ‘Distance’ and its interaction with ‘German language’ control for effects that happen away from the border and environmental differences. We restrict our analysis to municipalities within 100 km of the language border. Models (2), (3) and (4) include controls for the canton, referred to as Fixed Effects (FE). Models (3) and (4) include a control variable for municipality characteristics, whether the municipality is located in a rural or urban area. Model (4) includes additional controls at the municipality level. Controls include population age structure, average household size, birth rates, average revenue and tax rates. Robust standard errors are in parentheses. † *p* < 0.1; * *p* < 0.05; ** *p* < 0.01; *** *p* < 0.001. Source: Federal Statistical Office. Distances from search.ch.

**Table 4.** Revision of the Covid law: regression analysis at the language border

	(1)	(2)	(3) Baseline	(4)
German language	*−*0.0005	0.029^∗^	0.027^∗^	0.013
	(0.010)	(0.012)	(0.011)	(0.011)
German × Distance	0.001^∗∗∗^	*−*0.0003	*−*0.0004^†^	*−*0.001^∗^
	(0.0002)	(0.0003)	(0.0002)	(0.0002)
Distance	*−*0.002^∗∗∗^	*−*0.0002	*−*0.0002	0.0001
	(0.0002)	(0.0002)	(0.0002)	(0.0002)
Urban			0.094^∗∗∗^	0.079^∗∗∗^
			(0.005)	(0.005)
Constant	0.541^∗∗∗^	0.617^∗∗∗^	0.528^∗∗∗^	0.746^∗∗∗^
	(0.008)	(0.022)	(0.020)	(0.069)
Cantons FE	No	Yes	Yes	Yes
Municipality controls	No	No	No	Yes
Observations	1409	1409	1353	1353
Adjusted *R*^2^	0.186	0.272	0.440	0.489

Notes: The regression analysis shows the impact of switching from the French-speaking side of the border to the German-speaking side on voting results, that is, the proportion of ‘yes’ votes in a municipality. ‘German language’ indicates that the primary language of a municipality is German and is our variable of interest. ‘Distance’ is the road distance to the language border. ‘Distance’ and its interaction with ‘German language’ control for effects that happen away from the border and environmental differences. We restrict our analysis to municipalities within 100 km of the language border. Models (2), (3) and (4) include controls for cantons, referred to as Fixed Effects (FE). Models (3) and (4) include a control variable for municipality characteristics, whether the municipality is located in a rural or urban area. Model (4) includes additional controls at the municipality level. Controls include population age structure, average household size, birth rates, average revenue and tax rates. Robust standard errors are in parentheses. † *p* < 0.1; * *p* < 0.05; ** *p* < 0.01; *** *p* < 0.001. Source: Federal Statistical Office. Distances from search.ch.

### 3.2. Fertility-related referanda

#### 9 February 2014, referendum prohibiting the reimbursement of abortion

We now provide three examples related to fertility. We start with the referendum on the reimbursement of abortion. On 9 February 2014, Swiss citizens voted on the prohibition of the reimbursement of abortion by health insurance companies. Proponents of the proposal argued that taxpayers should not be forced to pay for a procedure they consider morally objectionable. Conversely, opponents argued that women should have access to safe and affordable abortion services, regardless of their financial situation.

**Figure 3.**
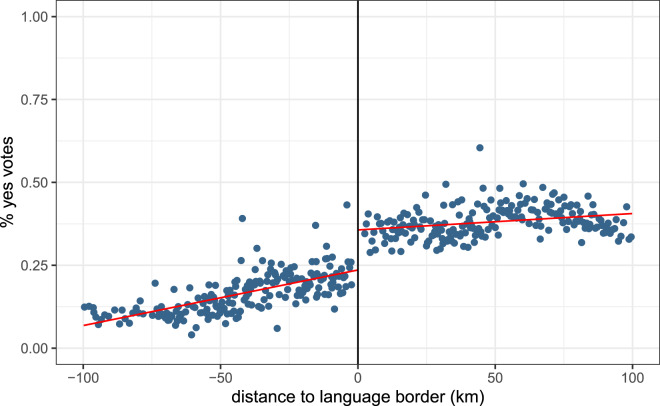
Average proportion of ‘yes’ votes to referendum prohibiting the reimbursement of abortion across municipalities, by distance to the language border. Notes: The left-hand side of the graph displays French-speaking municipalities; the right-hand side displays German-speaking municipalities. The red lines are the linear regression lines. Source: Federal Statistical Office. Distances from search.ch.

Figure 3 presents the percentage of votes in favour of the initiative across municipalities at different distances from the language border. The data show an evident discontinuity at the border. Municipalities on the French-speaking side of the border were less likely to vote in favour of modifying the law than municipalities on the German-speaking side. Regression analysis results in Table 5 confirm these descriptive results. The German language estimate is significant in the four models, and adding controls does not change this in any way (estimate = 0.127, *p <* 0.001).

**Table 5.** Referendum prohibiting the reimbursement of abortion: regression analysis at the language border

	(1)	(2)	(3) Baseline	(4)
German language	0.122^∗∗∗^	0.125^∗∗∗^	0.127^∗∗∗^	0.132^∗∗∗^
	(0.008)	(0.009)	(0.009)	(0.009)
German × Distance	*−*0.001^∗∗∗^	0.0003	0.0003^†^	0.0005^∗^
	(0.0002)	(0.0002)	(0.0002)	(0.0002)
Distance	0.002^∗∗∗^	0.0005^∗∗^	0.0005^∗∗^	0.0003^†^
	(0.0001)	(0.0002)	(0.0002)	(0.0002)
Urban			*−*0.041^∗∗∗^	*−*0.029^∗∗∗^
			(0.004)	(0.004)
Constant	0.236^∗∗∗^	0.133^∗∗∗^	0.174^∗∗∗^	*−*0.041
	(0.006)	(0.017)	(0.017)	(0.058)
Cantons FE	No	Yes	Yes	Yes
Municipality controls	No	No	No	Yes
Observations	1,409	1,409	1,353	1,353
Adjusted *R*^2^	0.676	0.756	0.770	0.786

Notes: The regression analysis shows the impact of switching from the French-speaking side of the border to the German-speaking side on voting results, that is, the proportion of ‘yes’ votes in a municipality. ‘German language’ indicates that the primary language of a municipality is German and is our variable of interest. ‘Distance’ is the road distance to the language border. ‘Distance’ and its interaction with ‘German language’ control for effects that happen away from the border and environmental differences. We restrict our analysis to municipalities within 100 km of the language border. Models (2), (3) and (4) include controls for cantons, referred to as Fixed Effects (FE). Models (3) and (4) include a control variable for municipality characteristics, whether the municipality is located in a rural or urban area. Model (4) includes additional controls at the municipality level. Controls include population age structure, average household size, birth rates, average revenue and tax rates. Robust standard errors are in parentheses. † *p* < 0.1; * *p* < 0.05; ** *p* < 0.01; *** *p* < 0.001. Source: Federal Statistical Office. Distances from search.ch.

Restricting women's access to abortion could have considerable genetic fitness implications, particularly for women. As cooperative breeders, mothers, and by extension fathers, require social support to raise their children. They must balance investment in their current offspring with investment in potential future offspring (Hrdy, 1999). In that sense, any restrictions on access to abortion would limit women's ability to manage this trade-off and impose a fitness cost on women. Acknowledging men's commitment to their offspring, these constraints and costs might not pertain to women only, but in some cases may extend to the couple. The 2014 referendum prohibiting the reimbursement of abortion in Switzerland could have resulted in such a cost, given the potential restrictions on access that the initiative could have imposed. Assuming that at the border one policy is better than the other in terms of average fitness, the discontinuity in the voting results suggests that one group was more willing to support a policy that would presumably impose an additional fitness cost on some individuals in the population relative to the other policy under consideration.

#### 5 June 2016, referendum on assisted reproduction

On 5 June 2016, Swiss citizens voted to modify the medically assisted reproduction law. The proposed amendment aimed to legalise, under certain conditions, the genetic diagnosis of embryos derived from *in vitro* fertilisation before implanting the embryos. The amended law would have allowed pre-implementation diagnosis only for carriers of alleles associated with severe hereditary disease or those who cannot have a child naturally. Supporters argued that the law was necessary to provide couples with the same reproductive options already available in neighbouring countries. On the other hand, opponents feared that the revision would have led to an ethically unacceptable expansion of genetic testing on human embryos and undermined the traditional family structure.

**Figure 4.**
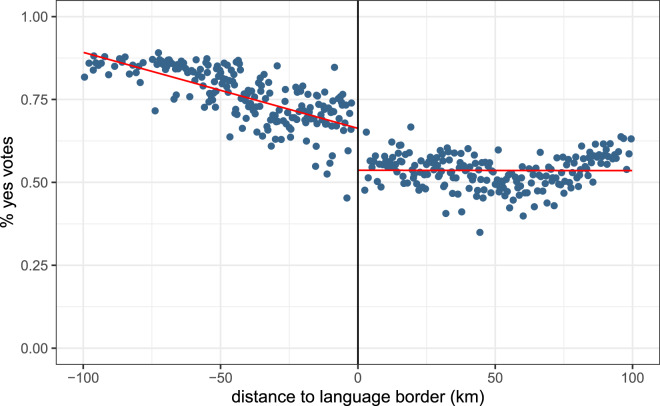
Average proportion of ‘yes’ votes to referendum allowing genetic diagnosis of embryos, across municipalities, by distance to the language border. Notes: The left-hand side of the graph displays French-speaking municipalities; the right-hand side displays German-speaking municipalities. The red lines are the linear regression lines. Source: Federal Statistical Office. Distances from search.ch.

Figure 4 shows the average proportion of ‘yes’ votes across municipalities at various distances from the language border. Data present a clear discontinuity at the border. Further, most data points on the French-speaking side of the border are above the data points on the German-speaking side. At the border, the French-speaking group is more likely to favour amending the law than the German-speaking group. These results are confirmed by the regression analysis results presented in Table 6. The German language estimate is significant and robust to additional municipality-level controls (estimate = *−*0.097, *p <* 0.001).

**Table 6.** Referendum on assisted reproduction: regression analysis at the language border

	(1)	(2)	(3) Baseline	(4)
German language	*−*0.131^∗∗∗^	*−*0.099^∗∗∗^	*−*0.097^∗∗∗^	*−*0.109^∗∗∗^
	(0.010)	(0.010)	(0.010)	(0.009)
German × Distance	0.002^∗∗∗^	*−*0.001^∗^	*−*0.001^∗∗^	*−*0.001^∗∗∗^
	(0.0002)	(0.0002)	(0.0002)	(0.0002)
Distance	*−*0.002^∗∗∗^	*−*0.0004^∗^	*−*0.0003^∗^	*−*0.0001
	(0.0002)	(0.0002)	(0.0002)	(0.0002)
Urban			0.050^∗∗∗^	0.033^∗∗∗^
			(0.004)	(0.004)
Constant	0.664^∗∗∗^	0.809^∗∗∗^	0.758^∗∗∗^	0.998^∗∗∗^
	(0.007)	(0.018)	(0.018)	(0.059)
Cantons FE	No	Yes	Yes	Yes
Municipality controls	No	No	No	Yes
Observations	1,409	1,409	1,353	1,353
Adjusted *R*^2^	0.638	0.772	0.792	0.820

Notes: The regression analysis shows the impact of switching from the French-speaking side of the border to the German-speaking side on voting results, that is, the proportion of ‘yes’ votes in a municipality. ‘German language’ indicates that the primary language of a municipality is German and is our variable of interest. ‘Distance’ is the road distance to the language border. ‘Distance’ and its interaction with ‘German language’ control for effects that happen away from the border and environmental differences. We restrict our analysis to municipalities within 100 km of the language border. Models (2), (3) and (4) include controls for cantons, referred to as Fixed Effects (FE). Models (3) and (4) include a control variable for municipality characteristics, whether the municipality is located in a rural or urban area. Model (4) includes additional controls at the municipality level. Controls include population age structure, average household size, birth rates, average revenue and tax rates. Robust standard errors are in parentheses. † *p* < 0.1; * *p* < 0.05; ** *p* < 0.01; *** *p* < 0.001. Source: Federal Statistical Office. Distances from search.ch.

The outcome of the 5 June 2016 referendum on pre-implantation genetic diagnosis could have had fitness consequences at the individual level. By allowing couples with serious hereditary diseases to implant healthy embryos selectively, the legalisations of pre-implantation diagnosis could have increased their offspring's chances of survival and reproduction, ultimately positively impacting individual fitness. However, genetic screening implies an opportunity cost. Using genetic screening for non-medical reasons, such as selecting specific traits such as eye colour or height, could result in a waste of resources. Unnecessary screening might divert limited resources away from other procedures that could matter more in terms of health. We do not know what screening level maximised individual fitness in that particular environment. Nonetheless, we observed that at the border the two groups had different preferences and associated voting behaviours. Assuming that at the border one policy is better than the other in terms of average fitness, supporting one policy would presumably impose a fitness cost on individuals relative to the other policy.

#### 27 September 2020, referendum on paternity leave

Our last example focuses on paternity leave. On 27 September 2020, Swiss citizens had to decide whether fathers should be granted two weeks of paid paternity leave. The proposed amendment to the Swiss Federal Constitution aimed to give fathers the right to take two weeks off work after the birth of a child. This leave would have been financed by the government. Proponents of the amendment argued that paternity leave would have provided fathers with the opportunity to bond with their newborns and help reduce gender inequality in the workplace and society. On the other hand, opponents claimed that the proposed paternity leave policy would have increased costs for employers and should not be legislated at the federal level.

**Figure 5.**
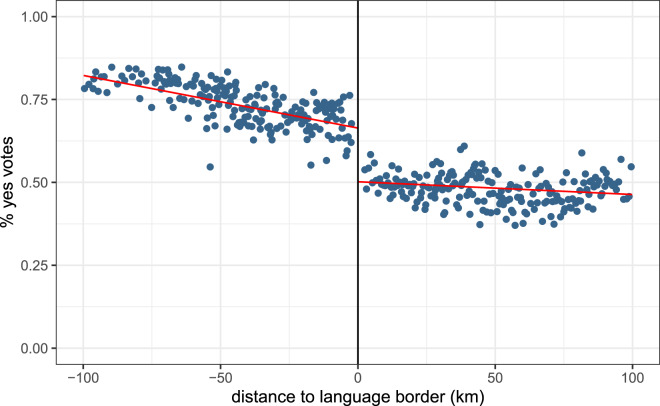
Average proportion of ‘yes’ votes to referendum on paternity leave across municipalities, by distance to language border. Notes: The left-hand side of the graph displays French-speaking municipalities; the right-hand side displays German-speaking municipalities. The red lines are the linear regression lines. Source: Federal Statistical Office. Distances from search.ch.

Figure 5 presents the average proportion of ‘yes’ votes for the referendum on paid paternity leave across municipalities at different distances from the language border. We observe an apparent discontinuity at the language border. Municipalities on the French-speaking side of the border were more likely to approve a paid paternity leave than those on the German-speaking side. Table 7 presents the regression analysis. The results confirm the descriptive evidence from the graph. The German language estimate is significant and not sensitive to additional controls (estimate = *−*0.160, *p <* 0.001).

**Table 7.** Referendum on paternity leave: regression analysis at the language border

	(1)	(2)	(3) Baseline	(4)
German language	*−*0.171^∗∗∗^	*−*0.155^∗∗∗^	*−*0.160^∗∗∗^	*−*0.159^∗∗∗^
	(0.009)	(0.010)	(0.009)	(0.010)
German × Distance	0.001^∗∗∗^	*−*0.0003	*−*0.0003	*−*0.001^∗^
	(0.0002)	(0.0002)	(0.0002)	(0.0002)
Distance	*−*0.002^∗∗∗^	*−*0.001^∗∗∗^	*−*0.001^∗∗^	*−*0.0003^†^
	(0.0001)	(0.0002)	(0.0002)	(0.0002)
Urban			0.067^∗∗∗^	0.058^∗∗∗^
			(0.004)	(0.004)
Constant	0.665^∗∗∗^	0.769^∗∗∗^	0.704^∗∗∗^	0.843^∗∗∗^
	(0.007)	(0.019)	(0.018)	(0.061)
Cantons FE	No	Yes	Yes	Yes
Municipality controls	No	No	No	Yes
Observations	1,409	1,409	1,353	1,353
Adjusted *R*^2^	0.715	0.773	0.811	0.822

Notes: The regression analysis shows the impact of switching from the French-speaking side of the border to the German-speaking side on voting results, that is, the proportion of ‘yes’ votes in a municipality. ‘German language’ indicates that the primary language of a municipality is German and is our variable of interest. ‘Distance’ is the road distance to the language border. ‘Distance’ and its interaction with ‘German language’ control for effects that happen away from the border and environmental differences. We restrict our analysis to municipalities within 100 km of the language border. Models (2), (3) and (4) include controls for cantons, referred to as Fixed Effects (FE). Models (3) and (4) include a control variable for municipality characteristics, whether the municipality is located in a rural or urban area. Model (4) includes additional controls at the municipality level. Controls include population age structure, average household size, birth rates, average revenue and tax rates. Robust standard errors are in parentheses. † *p* < 0.1; * *p* < 0.05; ** *p* < 0.01; *** *p* < 0.001. Source: Federal Statistical Office. Distances from search.ch.

Paternity leave may have had positive fitness consequences. Paternity leave allows fathers to spend more time with their newborn children. The more the father invests, the better the outcomes should tend to be for the current offspring. However, we could also imagine a countervailing effect for men. By investing time and resources in current offspring, fathers are potentially hindering their careers, which could make them less attractive in the future. Consequently, fathers are potentially hindering their ability to identify opportunities to mate with other women. In this sense, paternity leave could partially harm fathers’ fitness. We observe that the two groups adopted different voting behaviours at the border. Assuming the acceptance of paternity leave has fitness consequences and that at the border the optimal policy was the same, then one group showed stronger support for a policy that would have imposed fitness costs on some individuals compared to the other policy.

## 4. Discussion

We have investigated the causal influence of culture on health- and fertility-related choices using a spatial regression discontinuity design and Swiss referenda data. Our results show multiple discontinuities at the language border, especially with regard to fertility. Such discontinuities isolate cultural variation in preferences for policies that, if enacted, would have presumably affected health and fertility choices at the individual level. We have also speculated about connections between possible referenda outcomes and downstream effects on genetic fitness. Although the details of these speculations differ, the generic logic is always the same. For a given referendum, assume that one policy was better than the other policy in the sense that it would have promoted choices and created incentives that would have been better – in terms of individual expected fitness. We do not know which policy was better in this sense, but we assume that one was better, and the other was worse. If, in addition, the better policy right at the border was the same on both sides of the border, then any discontinuity in voting at the border implies that one of the two groups showed relative support for the worse policy for cultural reasons. More to the point, one of the two groups supported a policy that would have negatively affected health, survival and fertility relative to the other policy. By extension, the individuals in this group were ready to pay an opportunity cost in terms of fitness, and they were willing to impose this fitness cost on their Swiss fellows who would have been subject to the policy if enacted. We can view this opportunity cost in two ways. First, it would have represented an opportunity cost relative to the other policy under consideration. Second, it would have represented an opportunity cost in the form of reduced fitness relative to other societies, for example other countries in continental Europe.

While our findings emphasise cultural differences in health- and fertility-related voting decisions at the language border, our study comes with several limitations. First, our study employs municipal-level data and not individual-level data. This approach is well-suited for the central part of our analysis. We effectively demonstrate the capacity of culture to create stable differences between groups in domains related to health and fertility. However, this approach presents a significant limitation in exploring the potential impact of these cultural differences on variation in individual fitness values. Future research would benefit greatly by using individual-level data to assess more accurately how cultural differences affect the individuals who make up the cultural groups under study.

Second, individuals could have, in principle, self-selected into treatments. People born on the French-speaking side of the border could have moved to the German-speaking region in search of a cultural environment more aligned with their personal values and vice versa. Although we suspect that associated effects are trivial, we cannot definitively dismiss the potential impact of endogenous sorting into location at the border. Future research, equipped with more extensive data regarding the place of birth in lieu of the place of residence, would be better poised to control for any possible selection bias of this sort. Third, our sample consists solely of voters and is thus unrepresentative of the Swiss population. That said, laws and policies are enacted precisely on the basis of the preferences and decisions of voters, and in this sense our sample represents the politically engaged part of the population. As such, our data demonstrate how culture can shape voting decisions and policy outcomes.

Fourth, we do not know how cultural variation in voting translates into cultural variation in behaviour. For instance, we found clear distinctions in voting about paternity leave. Yet we do not know how these kinds of differences might relate to the time fathers spend with their children, and we do not know how people on both sides of the border might react to one policy vs. another. In general, we can imagine that the two groups might often support different policies, but they might also react differently to the policy that prevails after all the votes are tallied. Future research could examine these kinds of questions by exploring cultural differences in behavioural responses to political outcomes.

Finally, the data only pertain to referenda results and do not distinguish between the different reasons people vote one way or another. Our task was to isolate, as much as possible, the effects of culture from the effects of environments, institutions and even genes. Our approach separates the influence of culture on voting in this way, but it cannot identify which components of culture drive results. Similarly, we cannot control for variation in the social environment. Observed variation at the border could be driven by differences in cultural domains related to religion, political affiliation, media consumption or secular values. Future studies could unpack the discontinuities by investigating these kinds of underlying mechanisms.

Within the boundaries of these limitations, we have attempted to add a crucial element to the discussion of gene–culture processes by pushing for the clean identification of culture as a distinct cause of health- and fertility-related choices. In particular, genetic evolutionary processes do not favour stable differences between groups. Minimal gene flow between groups is enough to render groups nearly identical genetically (Bell et al., 2009; Frankham et al., 2002), and this seems to be the state of affairs at the Röstigraben in Switzerland (Buhler et al., 2012). This is crucial because, if groups are genetically similar, selection at the group level is irrelevant. If groups are different, in contrast, selection at the group level could easily matter. In this latter case, group selection can shape evolutionary dynamics in addition to selection at the individual level, and the result can be entirely new evolutionary regimes that would not otherwise be possible. Although the workaday evolutionary ecologist generally ignores such possibilities in strictly genetic systems, cultural evolutionary processes may be completely different (Mesoudi & Danielson, 2008; Richerson et al., 2016). Our results show that cultural evolution can stabilise differences between groups, even amid ongoing contact, and it can do so in decision-making domains that should have a relatively close link to genetic fitness.

In particular, under the assumption that fitness effects are equivalent right at the border on both sides of the border, our results suggest that voters on one side or another routinely support a policy that was worse in terms of expected fitness than the other policy under consideration. The policy is worse in the sense that it should impose a cost in terms of expected fitness on individuals subject to the policy, but support for the policy is to some extent a group-level cultural phenomenon. This suggests the potential for cultures to maintain preferences detrimental to fitness when compared with some relevant benchmark. However, there are exceptions where this assumption does not hold – cases with complex fitness landscapes where multiple equivalent optima exist and variations in social environments that affect policy fitness consequences. These scenarios are crucial for a comprehensive understanding, yet they complement rather than contradict our primary observation. Cultural influences have the potential to shape preferences in ways that may not always align with optimal fitness outcomes.

These results are especially surprising because they hold in contemporary Switzerland. Switzerland is one of the easiest places in the world to get from one place to another. The distances are short, and the trains are clean, pleasant, frequent, extremely long and exceedingly reliable. Moreover, this has been the state of affairs for a long time. The flow of cultural information across the border on a daily basis must be extreme, and thus one might naively expect the Röstigraben to be a cute vestige of former times. Our results, however, show that the reality is quite the opposite.

Altogether, given the limitations of our approach, our contribution is twofold. First, we highlight the value of using a quasi-experimental design to isolate the causal influence of culture on decision making. Strangely, many of us are probably comfortable with the notion that somehow cultural differences exist. However, from a strictly empirical perspective, cultures routinely covary with other confounds, and separating the effects of culture from these confounds can often be difficult or impossible. Our approach does so by essentially identifying systematic group-level differences that cannot be genetic, environmental or institutional. Second, we specifically isolate cultural effects of this sort in decision-making domains related to health and fertility. In this way, although we do not examine genetic fitness directly, we do lean in this direction by focusing on cultural variation in support of policies that should influence fertility, health and survival. The variation in question is a group-level phenomenon based on cultural evolutionary processes, but it should have consequences for individual reproduction and by extension fitness.

## Supporting information

Faessler et al. supplementary material 1 Faessler et al. supplementary material

Faessler et al. supplementary material 2 Faessler et al. supplementary material

## Data Availability

The data supporting the findings of this study are available within the supplementary materials. Please visit https://doi.org/10.1017/ehs.2024.19.

## Supplementary material

To view supplementary material for this article, please visit https://doi.org/10.1017/ehs.2024.19

## Research transparency and reproducibility

The data used in this study are publicly available at https://www.bfs.admin.ch/. Datasets, including distance data, and codes for analysis are available in the supplementary material.
